# Complex Population Structure of Lyme Borreliosis Group Spirochete *Borrelia garinii* in Subarctic Eurasia

**DOI:** 10.1371/journal.pone.0005841

**Published:** 2009-06-09

**Authors:** Pär Comstedt, Loreta Asokliene, Ingvar Eliasson, Björn Olsen, Anders Wallensten, Jonas Bunikis, Sven Bergström

**Affiliations:** 1 Department of Molecular Biology, Umeå University, Umeå, Sweden; 2 Department of Infectious Diseases, Dermatovenerology and Microbiology, Vilnius University, Vilnius, Lithuania; 3 Department of Clinical Microbiology and Immunology, Lund University Hospital, Lund, Sweden; 4 Section of Infectious Diseases, Department of Medical Sciences, Uppsala University, Uppsala, Sweden; 5 Section for Zoonotic Ecology and Epidemiology, School of Pure and Applied Natural Sciences, University of Kalmar, Kalmar, Sweden; St. Petersburg Pasteur Institute, Russian Federation

## Abstract

*Borrelia garinii*, a causative agent of Lyme borreliosis in Europe and Asia, is naturally maintained in marine and terrestrial enzootic cycles, which primarily involve birds, including seabirds and migratory passerines. These bird groups associate with, correspondingly, *Ixodes uriae* and *Ixodes ricinus* ticks, of which the latter species may bite and transmit the infection to humans. Studies of the overlap between these two natural cycles of *B. garinii* have been limited, in part due to the absence of representative collections of this spirochete's samples, as well as of the lack of reliable measure of the genetic heterogeneity of its strains. As a prerequisite for understanding the epidemiological correlates of the complex maintenance of *B. garinii*, the present study sought to assess the diversity and phylogenetic relationships of this species' strains from its natural hosts and patients with Lyme borreliosis from subarctic Eurasia. We used sequence typing of the partial *rrs-rrl* intergenic spacer (IGS) of archived and prospective samples of *B. garinii* from *I. uriae* ticks collected predominantly on Commander Islands in North Pacific, as well as on the islands in northern Sweden and arctic Norway. We also typed *B. garinii* samples from patients with Lyme borreliosis and *I. ricinus* ticks infesting migratory birds in southern Sweden, or found questing in selected sites on the islands in the Baltic Sea and Lithuania. Fifty-two (68%) of 77 *B. garinii* samples representing wide geographical range and associated with *I. ricinus* and infection of humans contributed 12 (60%) of total 20 identified IGS variants. In contrast, the remaining 25 (32%) samples recovered from *I. uriae* ticks from a few islands accounted for as many as 10 (50%) IGS types, suggesting greater local diversity of *B. garinii* maintained by seabirds and their ticks. Two IGS variants of the spirochete in common for both tick species were found in *I. ricinus* larvae from migratory birds, an indication that *B. garinii* strains are exchanged between different ecological niches. Notably, *B. garinii* variants associated with *I. uriae* ticks were found in each of the six clusters, representing two phylogenetic lineages of this species identified among the studied samples. Our findings suggest that *B. garinii* in subarctic Eurasia comprises two partially overlapping populations with different levels of genetic heterogeneity, presumably, due to distinctive selective pressures on the spirochete in its marine and terrestrial enzootic cycles.

## Introduction

Lyme borreliosis (LB) is the most prevalent arthropod-born disease in Europe and North America. The infection is caused by LB group *Borrelia* genospecies, which are transmitted by *Ixodes* ticks. *Borrelia burgdorferi* sensu lato (further *B. burgdorferi*) is the LB agent in North America, whereas this species, *Borrelia afzelii* and *Borrelia garinii* cause the disease in Eurasia.

Major natural reservoirs of *Borrelia* spp. are small mammals and birds. In Europe, where *Ixodes ricinus* ticks transmit the spirochetes, rodents are the primary hosts of *B. afzelii*. *B. garinii* is preferentially associated with bird reservoirs, including pheasants in the United Kingdom and migratory passerines on the continent [1]–[3]. The latter bird group, with major migratory routes along the coastlines with high human population density, both disseminates infected ticks and is a reservoir of *B. garinii*, including strains causing infection in humans [1].

In addition to *B. garinii*'s terrestrial natural cycle, we have previously demonstrated this species' association with marine birds and their common globally distributed tick *Ixodes uriae* [4]. Presumably due to migration of many seabird species over great distances, closely related *B. garinii* isolates have been found in *I. uriae* ticks in the northern and the southern hemisphere [5]–[7]. *B. garinii* 's association with the seabirds expands the habitat range of this species even further to the costal regions in the northern hemisphere, including both the eastern and western coasts of North America [7], [8]. On the other hand, the prevalence of *Borrelia* spirochetes among seabirds and their ticks in the northern Pacific Ocean is unknown. The islands in this part of the Pacific Ocean have a number of large seabird colonies and the east Pacific flyway for migrating birds stretches along the East Asian coast [9].

*B. garinii* is thought to be genetically and antigenically most heterogeneous species among LB group genospecies. Typing with monoclonal antibodies specific for the outer surface protein A (OspA) has revealed that *B. garinii* accounts for 5 of total 7 OspA serotypes found among LB group genospecies [10]. This first indication of extensive antigenic heterogeneity of *B. garinii* was further confirmed by identifying sub-variants among this species' OspA serotypes [11]. Studies of other genetic loci of this spirochete's strains from the natural and clinical sources have also demonstrated broad antigenic and genetic diversity [10], [12]–[14]. However, no attempt has yet been made to compare the diversity of *B. garinii* from different ecological niches and biological sources, in part, because of the lack of consensus about genetic approaches for typing its strains.

To further understand the relationship between the marine and terrestrial enzootic cycle of *B. garinii*, in the present study we compared genetic variability of this species' strains primarily from *I. uriae* and *I. ricinus* tick collections in subarctic Eurasia. We found that *B. garinii* in this geographical region comprises two partially overlapping populations with differing genetic diversity.

## Materials and Methods

### The site and field collections

**Figure 1:**
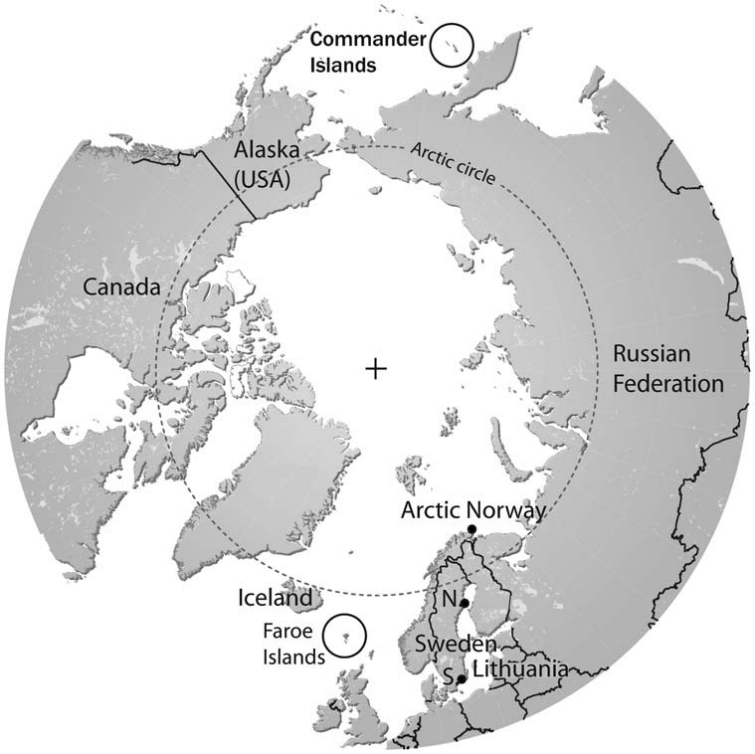
Map of the Arctic and Subarctic regions. Studied *B. garinii* isolates originated from selected locations in southern Sweden (indicated with “S.” and a filled circle), northern Sweden (indicated with “N.” and a filled circle), arctic Norway (filled circle), Commander Islands and Faeroe Islands (encircled), and Lithuania.

The Commander Islands are located in the North Pacific Ocean, and are bordered to the north by the Bering Sea. They are situated approximately 175 kilometers to the east of the Kamchatka Peninsula of the Russian Federation (Figure 1). The map of the Arctic and subarctic region was obtained with permission from UNEP/GRID-Arendal Maps and Graphics Library at http://maps.grida.no/go/graphic/arctic-map-political. All ticks were collected on the 5^th^ and 6^th^ of July 2007, from the ground in a Tufted Puffin (*Lunda cirrhata*) colony located on Toporkov (in Russian, “puffin”) Island (55°12'N, 165°56E) 4 km West of the main, Bering, island. Toporkov Island is flat, 0.5 square km in size, with Tufted Puffin colony occupying most of its surface. About 100 gulls and a few cormorants also nest on the beach and outermost cliffs, respectively. No mammals are present on the island (personal communication with personnel at Komandorskiy State Nature Biosphere Reserve).

Questing or engorged *I. uriae* larvae, nymphs and adults were collected from different locations of the island. The birds were caught using landing nets and, in addition to the ticks, the blood was drawn from the tarsal vein by syringe and a thin needle. The geographical origin and biological source of this and the previous collections of the ticks, included in the study, are presented in Table 1.

**Table 1:** Geographical and biological origin and designation* of genetic variants of studied *B. garinii* samples.

Genetic variant*	No. samples	Phylo-genetic cluster	Sample(s) name	Biological origin	Geographical origin	Reference
1	5	2	Var1	*I. uriae* from Guillemot colony	Arctic Norway	[6]
			Mal01	*I. uriae* from Black Guillemot.	N. Sweden	[5]
			Far01, Far02,	*I. uriae* from Puffin colony.	Faroe Islands	[23]
			Far04	Puffin Blood	Faroe Islands	[23]
2	2	6	K22	*I. ricinus* nymph from European robin	Mig. S. Sweden	[1]
			Bio56002	Human skin	S. Sweden	[38]
3	1	6	Var3	*I. uriae* from Guillemot colony	Arctic Norway	[6]
4	5	6	A15, C55, G09	*I. ricinus* nymph from Black bird, Thrush nightingale, Song thrush	Mig. S. Sweden	[1]
			Bio56014, Bio56045	Human skin	S. Sweden	[38]
5	6	4	C24, D49, E07	*I. ricinus* nymph from Redstart, Black bird thrush nightingale	Mig. S. Sweden	[1]
			Bio56016, Bio56081	Human skin	S. Sweden	[38]
			Lit27	*I. ricinus*	Lithuania	This study
6	6	4	B02	*I. ricinus* nymph from Redstart	Mig. S. Sweden	[1]
			D12	*I. ricinus* larva from Tree pipit	Mig. S. Sweden	[1]
			Bio56056, Bio30058, Bio56101	Human skin	S. Sweden	[38]
			Lit25	*I. ricinus*	Lithuania	This study
7	1	4	Var2	*I. uriae* from Guillemot colony	Arctic Norway	[6]
8	3	5	Com42, Com99, Com293	*I. uriae* from Tufted puffin colony	Com. Isl. Russia	This study
9	2	5	Com65, Com329	*I. uriae* from Tufted puffin colony	Com. Isl. Russia	This study
10	2	3	Com96, Com261	*I. uriae* from Tufted puffin colony	Com. Isl. Russia	This study
11	5	1	Com72, Com82, Com84, Com92, Com235	*I. uriae* from Tufted puffin colony	Com. Isl. Russia	This study
12	4	1	Com22, Com32, Com81, Com341	*I. uriae* from Tufted puffin colony	Com. Isl. Russia	This study
13	12	3	A99, D46, D48, D88	*I. ricinus* larvae from Great tit, Black bird, Tree pipit, Tree pipit	Mig. S. Sweden	[1]
			E09, F88	*I. ricinus* nymph from Black bird, Song thrush	Mig. S. Sweden	[1]
			Bio56059, Bio56061	Human skin	S. Sweden	[38]
			Lu116	Human skin	S. Sweden	[39]
			Lu190	Human cerebrospinal fluid	S. Sweden.	[39]
			Lit72, Lit89	*I. ricinus*	Lithuania	This study
14	2	2	Mal02	*I. ricinus*	N. Sweden	[5]
			NBS47	*I. ricinus*	N. Sweden	[5]
15	1	6	Lit 20	*I. ricinus*	Lithuania	This study
16	4	5	Var4	*I. uriae* from Guillemot colony	Arctic Norway	[6]
			NBS49	*I. ricinus*	N. Sweden	[5]
			C78	*I. ricinus* larva from Starling	Mig. S. Sweden	[1]
			E08	*I. ricinus* nymph from Tree pipit	Mig. S. Sweden	[1]
17	4	2	IUB18	*I. uriae* from Razorbill	N. Sweden	[4]
			NBS16	*I. ricinus*	N. Sweden	[4]
			C51	*I. ricinus* larva from Black bird	Mig. S. Sweden	[1]
			Lit44	*I. ricinus*	Lithuania	This study
18	4	3	D83, F89	*I. ricinus* nymph from tree pipit, Song thrush	Mig. S. Sweden	[1]
			LU118	Human skin	S. Sweden	[39]
			Lu222	Human cerebrospinal fluid	S. Sweden	[39]
19	5	3	A35, B69	*I. ricinus* nymph from Song thrush, European robin	Mig. S. Sweden	[1]
			Bio56077	Human skin	S. Sweden	[38]
			Lu59	Human cerebrospinal fluid	S. Sweden	[39]
			Lit 42	*I. ricinus*	Lithuania	This study
20	3	3	NBS23B	*I. ricinus*	N. Sweden	[29]
			D40	*I. ricinus* larva from Black bird	Mig. S. Sweden	[1]
			D47	*I. ricinus* nymph from Black bird	Mig. S. Sweden	[1]

* Designation is based on partial *rrs-rrl* intergenic spacer sequence-typing (see in the text and Figure 3).

Abbreviations: Com. Isl, Commander Islands; Mig. migrating; N. Northern; S. Southern.

### *Borrelia* cultures from ticks

Ticks were washed in 70% ethanol and cut in halves with a scalpel. One half was put in 5 ml BSKII medium supplemented with 9% rabbit serum (Sigma) fosfomycin (100 µg/ml) and sulfamethoxazole (50 µg/ml), and incubated at 35°C. The cultures were screened for the growth of *Borrelia* spirochetes by phase contrast microscopy. Another half of the tick was subjected to DNA extraction as described below.

### Quantitative PCR on ticks and blood from birds

DNA was extracted from 299 ticks and 86 bird-blood clots using DNeasy Blood and Tissue Kit (Qiagen) according to the manufacturer's instructions, except for an initial overnight incubation at 60°C with Proteinase K (Roche) at 1.44 µg/µl final concentration. The DNA extracts were assayed for LB group spirochetes, using quantitative real time PCR (qPCR) assay with the probe and primers specific for the 16S rRNA gene of *Borrelia* species, as previously described [15]. Briefly, the forward and reverse primers at 900 nM were, respectively, 5 –GCTGTAAACGATGCACACTTGGT and5 -GGCGGCACACTTAACACGTTAG. The corresponding dye-labeled probes at 200 nM were 6FAM-TTCGGTACTAACTTTTAGTTAA and VIC-CGGTACTAACCTTTCGATTA modified with a minor groove binding (MGB) protein (Applied Biosystems). The PCR conditions were 50°C for 2 min and 95°C for 10 min, followed by 45 cycles of 95°C for 15 s and 63°C for 60 s. For standards, control DNA was extracted from *B. burgdorferi* B31 and *Borrelia hermsii* HS1 and serially diluted as described [15]. To assess the inhibitory effect of the blood DNA extracts on the qPCR, control blood samples were spiked with a known number of *B. burgdorferi* B31 spirochetes prior to DNA extraction.

### Identification of *Borrelia* species and genetic variants

**Figure 2:**
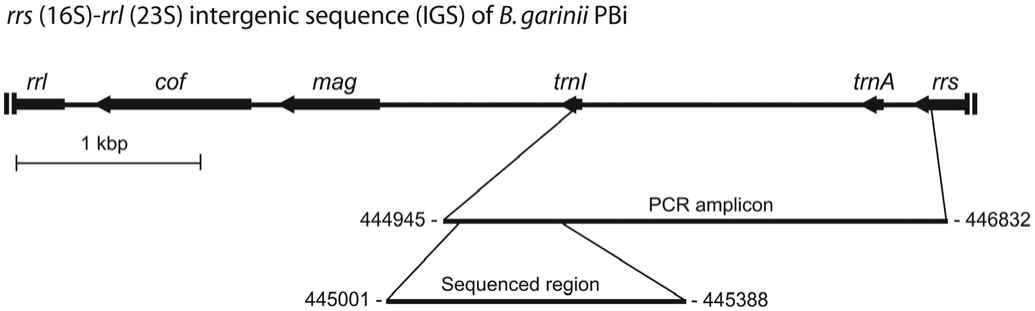
Physical map of partial rRNA operon of *B. garinii* strain PBi. In the operon, the *rrs-rrl* intergenic spacer (IGS) separates the *rrs* (16S) gene from *rrl* (23S) gene on the chromosome. The forward primers for the IGS amplification by nested PCR are at the 3′ end of the *rrs* and the reverse primers are in the *trnI* gene. Included in the IGS amplicon is the *trnA* gene. The *mag* and *cof* genes of the IGS region are located downstream of the PCR target region. The nucleotide positions for the 5′ and 3′ ends of the PCR amplicon and of the sequenced region are shown; numbering follows the coordinates of *B. garinii* PBi chromosome available under GenBank accession number CP000013 [28]. The scale of the map in kilobasepairs (kbp) is indicated.

*Borrelia* species was identified by direct sequencing of the amplicons generated by nested PCR of partial *rrs* (16S)-*rrl* (23S) intergenic spacer (IGS) region, as previously described [16]. The nested forward primer 5′AGGGGGGTGAAGTCGTAACAAG for the partial IGS locus was at the 3′ end of the *rrs* gene, and the nested reverse primer 5′GTCTGATAAACCTGAGGTCGGA was in the coding sequence of *trnI* gene for the tRNA-Ile in the spacer (Figure 2). Sequences were initially aligned using the CLUSTAL X algorithm [17], and then manually using MacClade 4.04 software [18]. Positions with at least two different characters in at least two sequences each were considered polymorphic, and included in the analyses. With few exceptions, singletons, i.e. variant nucleotides found in only one sequence, were ignored. Descriptive statistics of the aligned sequences was carried out with version 3.5 of the DnaSP suite of programs [19]. GENECONV version 1.81 (www.math.wustl.edu/~sawyer/mbprogs) was used to perform Sawyer's test for evidence of gene conversion; it examines the null hypothesis that nucleotide substitutions observed in a set of aligned sequences are randomly distributed [20]. The implementation of Jolley et al. [21] in their START suite of algorithms (http://pubmlst.org/software/analysis/start/) of the maximum chi-squared test of Maynard Smith was used to identify possible recombination events between pairs of alleles; the significance level (*p* value) for each pair-wise analysis was the proportion of 1000 permutations that had maximum chi-squared values greater than or equal to the observed chi-squared value. Phylogenetic analysis was performed on aligned sequences, without modification or character weight change, using neighbour-joining, maximum parsimony or maximum-likelihood methods of the PHYLO_WIN phylogenetic analysis program [22]. Percentage support values for clades were obtained from 100 bootstrap iterations in maximum-likelihood and 1000 iterations in other routines.

## Results

### Prevalence of *Borrelia* infection of *I. uriae* ticks

Our previous studies on *B. garinii* infection of *I. uriae* ticks associated with the seabirds in subarctic region have produced a limited number of this spirochete's samples (Table 1) [4], [5], [23]. In order to carry out a more comprehensive analysis of both local and regional populations of *B. garinii* in the marine ecological niche, we sought to expand this species' collection from *I. uriae* ticks. In total, about 700 ticks were collected from the ground in the Tufted Puffin colony on one of the Commander Islands in the North Pacific. The tick collection comprised 5 different groups: engorged larvae, questing and engorged nymphs, and questing and engorged adults (Table 2). Of the 299 ticks tested by qPCR, 99 (33%) were positive for LB group *Borrelia* spirochetes. The infection prevalence among questing adults was 30 (40%) of 75, engorged larvae 24 (36%) of 66, engorged nymphs 14 (29%) of 47, engorged adults 15 (29%) of 52, and questing nymphs 16 (27%) of 59. There was no significant difference in the prevalence of infection between these groups of ticks (Fisher's exact test *P* value>0.05).

**Table 2:** The prevalence and counts of *B. garinii* in *I. uriae* ticks from Commander Islands, Russia.

Tick sample	No. tested	No. positive (%)	Mean cell counts (95% CI)*
Engorged larvae	66	24 (36)	2123 (673–6698)
Questing nymphs	59	16 (27)	16 (5–49)
Engorged nymphs	47	14 (30)	893 (144–5546)
Questing adults	75	30 (40)	209 (54–807)
Engorged adults	52	15 (29)	2489 (535–11561)
Total	299	99 (33)	n.a.

* Mean and asymmetric confidence intervals (CI) are antilogs of log_10_-transformed data from positive samples. n.a., not applicable.

### Quantification of *Borrelia* infection of natural hosts

LB group spirochetes are sensitive to host blood components, which is one determinant of their preferential association with the vertebrate host species [2]. Therefore, spirochete proliferation in an engorging infected nymphal or adult tick is indicative of the mammalian or bird host's competence in facilitating the transmission of the infection [1], [24]. We next sought to assess Tufted Puffins' reservoir role by comparing spirochete counts in infected *I. uriae* ticks at different developmental and feeding stages.

In engorged nymphal and adult *I. uriae*, mean spirochete counts were significantly greater as compared to the corresponding stages of questing ticks: 893 (95% CI 144–5546) versus 16 (5–49) spirochetes per infected nymph (*P*<0.001) and 2489 (535–11561) versus 209 (54–807) per infected adult (*P* = 0.015) (Table 2). Engorged larvae were infected at mean cell density similar to the numbers found in other engorged tick stages, suggesting efficient transmission of infection from the birds to larvae. It was apparent, however, that molting from engorged larvae to questing nymphs significantly reduced the mean bacterial density, from 2123 (673–6698) to 16 (5–49) per infected tick (*P*<0.0001). A similar trend was noted also for engorged nymphs molting to questing adults: 893 (144–5546) versus 209 (54–807) spirochetes per infected tick, respectively (*P* = 0.20).

None of the 86 blood samples from Tufted Puffins was positive for *Borrelia* spirochetes by qPCR. In the experiment with selected blood DNA extracts spiked with *Borrelia* DNA to control for possible inhibition of qPCR assay by blood extract components, all samples were positive by the assay.

### Genetic diversity of *B. garinii*

*Borrelia* species and genotypes associate with their vertebrate reservoir hosts with overlapping specificity [2], [25]. With respect to *B. garinii*, which preferentially associates with birds [3], [4], [26], [27], genetic variation at the species level may be a marker of selective pressure to these spirochetes from their hosts' immune system. To advance the understanding of the complexity of *B. garinii* maintenance in nature, we next typed *B. garinii* samples from diverse biological and geographical sources (Table 1).

We performed sequence typing of this spirochete's partial *rrs* (16S)*-rrl* (23S) IGS locus, which is an effective typing marker of the strains of both Lyme borreliosis and relapsing fever group *Borrelia* species [16]. The criterion for designating a distinct variant, e.g. variant 1 of *B. garinii* (Table 1), was the presence of a unique set of sequence polymorphisms at the aligned region of the *rrs-rrl* IGS locus. The nested forward primer for the partial IGS locus was at the 3′ end of the *rrs* gene, and the nested reverse primer was in the coding sequence of *trnI* gene for the tRNA-Ile in the spacer. The amplicon included the *trnA* gene for tRNA-Ala, and varying lengths, from about 500 bp to 1.8 kbp, of the total 4617 bp IGS of *B. garinii* by the PBi strain coordinates (GenBank accession number CP000013) (Figure 2). The *mag* gene for methyladenine DNA glycosylase and the gene for *cof* hydrolase were situated downstream of the amplified IGS region [28].

Table 3 presents the descriptive statistics on the alleles of the analyzed region of the *rrs-rrl* IGS locus of *B. garinii*, which corresponds to the positions from 445002 to 445388 of CP000013. For comparison, the table includes also previously reported findings for the overlapping region of the partial *rrs-rrl* IGS locus of *B. burgdorferi* and *B. afzelii* [16]. Among the sequences from 77 *B. garinii* samples of cultured isolates or extracts of infected ticks, there were 20 sequence variants of the IGS region that ranged from 388 to 393 nucleotides in length. For non-gapped positions, the mean nucleotide diversity per position (π) was 0.056, or 2- and 4-fold greater than π-value for *B. burgdorferi* and *B. afzelii rrs-rrl* IGS, respectively (Table 3). The diversity was the greatest (π = 0.06) among the 10 alleles identified among *B. garinii* samples from either exclusively *I. uriae* ticks (8 variants) or found in common for *I. uriae* and *I. ricinus* ticks (2 variants). This was followed by π = 0.048 for the 12 alleles identified in a group of samples associated with *I. ricinus* ticks. Notably, the polymorphism at the IGS region for the 7 variants of *B. garinii* found in the patients with Lyme borreliosis was 8%, which was about two-fold lower than allelic heterogeneity of the spirochete's variants found in *I. uriae* or *I. ricinus*.

**Table 3:** Descriptive statistics and Sawyer's test for recombination of partial *rrs-rrl* intergenic spacer region of *B. garinii* samples from subarctic Eurasia.

*Borrelia* species and sample group	No. samples	No. alleles	Aligned characters				Sawyer test			
			Base pairs	No. gapped	Polymor-phisms (%)	π	Max. score	SD	*p*-value	Significant fragments
***B. garinii***	77	20	393	21	92 (23)	0.056	7.23	3.60	0.003	0
I. uriae and I. ricinus	31	10	392	17	70 (18)	0.060				
I. ricinus	52	12	388	15	63 (16)	0.048				
I. ricinus and patients	40	7	388	15	32 (8)	0.035				
***B. burgdorferi*** 1	68	24	812	11	60 (7)	0.025	2.7	−0.34	0.57	0
***B. afzelii*** 1	107	11	400	0	17 (4)	0.013	3.1	2.59	0.02	0

π, mean nucleotide diversity at each aligned position.

SD, number of standard deviations above the mean of 10000 permutations using GENECONV.

*p*-value, simulated *p* value based on 10000 permutations with Bonferroni correction for multiple samples.

Significant fragments, number of inner fragments with Bonferroni-corrected Karlin-Altschul *p* values of<0.05.

1 Included for comparison from Bunikis et al. [16].

### *B. garinii* population structure

*B. garinii* genetic variation study produced *rrs-rrl* IGS sequence marker for measuring the relationships between this spirochete's variants across wide geographical range of subarctic Eurasia. Examples of such relationships could be an overlap and exchange of the spirochete's circulation among diverse tick species and vertebrate reservoirs, including seabirds, migratory birds and, possibly, mammalian hosts [1], [5], [23]. As a prerequisite for understanding these associations, as well as the epidemiological and clinical correlates of *B. garinii* diversity, we next assessed this spirochete's population structure among the studied samples.

Most of the 20 *B. garinii* variants identified among the 77 samples were represented by at least two occurrences (Table 1). The exceptions, for which only a single sample was found, were *B. garinii* variants 3 and 7 from *I. uriae* ticks in a guillemot colony in arctic Norway, and the variant 15 identified in a questing *I. ricinus* nymph in Lithuania. The 16 samples of *B. garinii* isolated from questing *I. uriae* ticks on Commander Islands produced as many as 5 IGS sequence types (variants 8 through 12; Table 1). Furthermore, 9 samples found in *I. uriae* ticks from arctic Norway, Faroe Islands in northern Atlantic or northern Sweden formed 5 additional IGS types (variants 1, 3, 7, 16 and 17). Overall, 25 *B. garinii* samples recovered from *I. uriae* ticks, or 32% of the 77 total samples, comprised 10 (50%) of the 20 IGS types.

In contrast to the extensive IGS diversity found among *B. garinii* samples from *I. uriae*, the most prevalent IGS type among the samples from other sources, variant 13, included as many as 12 samples. These samples represented the geographical range from Sweden to Lithuania and the biological origin as diverse as questing or migratory bird-infesting *I. ricinus* ticks and patients with Lyme borreliosis. Similarly, an aggregate of other 17 samples with analogous geographical distribution and biological sources produced only 3 additional IGS types (variants 5, 6 and 19).

We found two examples of a possible sharing of *B. garinii* variants between the ecological cycles involving *I. uriae* and *I. ricinus* ticks. In the first case, *B. garinii* variant 16 was identified in *I. uriae* ticks collected in guillemot colony in arctic Norway, as well as in *I. ricinus* larva and nymph from migratory passerine birds captured in southern Sweden (Table 1) [1]. In another instance, *B. garinii* variant 17 infected *I. uriae* and *I. ricinus* ticks on nearby islands in the Gulf of Bothnia [4], [29] of the Baltic Sea, as well as the *I. ricinus* larva from a migratory bird and a questing nymph in Lithuania. Importantly, the infection of *I. ricinus* larvae from migratory birds with *B. garinii* variants shared by the two tick species indicates not only a disseminator, but also a reservoir role of these birds in the spirochete's natural maintenance.

### Phylogeny of *B. garinii* genotypes from subarctic Eurasia

In order to investigate the evolutionary origins of *B. garinii* genetic variants, we initially evaluated the aligned *rrs-rrl* IGS sequences for evidence of intragenic recombination, which could confound attempts to identify monophyletic groups. Sawyer's test assesses the likelihood for a set of aligned homologous sequences that the polymorphic fragments arose through recombination rather than mutation [20]. The test is appropriate for sets of sequences with the level of nucleotide diversity shown by the IGS sequences [16], which have a sufficient number of informative polymorphic sites. As summarized in Table 3, the Sawyer's test found no evidence of recombination at the IGS locus of *B. garinii* strains. By this test there were two significant inner fragments detected among the IGS sequences with simulation *p* = 0.003, but these were rejected by the more conservative Karlin-Altschul criteria. In addition, the maximum chi-squared test was applied to the 372 non-gapped positions of the 20 IGS sequence types of *B. garinii*. Even without Bonferroni correction, the *p* values were >0.10 for all 190 pairs.

**Figure 3:**
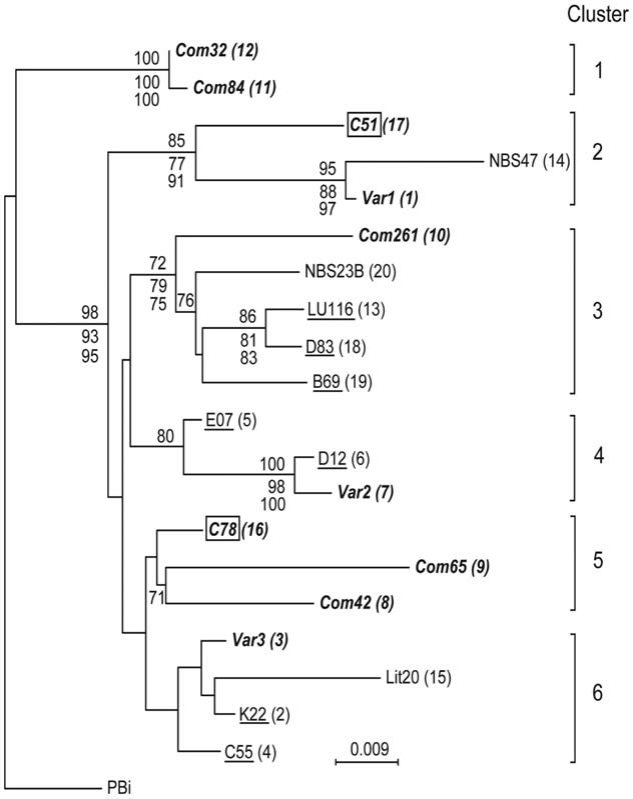
Distance phylogram from nucleotide sequence alignment of partial *rrs-rrl* IGS of *B. garinii* variants of diverse geographical and biological origin. The names of the representative samples are used for taxa designation in the tree, with the corresponding variant number, according to the list in Table 1, indicated in parenthesis. Bootstrap values for nodes with at least 70% support by neighbor-joining distance (1,000 replicates; number above the line) and/or maximum-likelihood (100 replicates; first number below the line) and maximum parsimony (1,000 replicates; second number below the line) criteria are shown. The tree is based on observed divergence option of distance method in PHYLO_WIN. A maximum-likelihood reconstruction applied transitions/transversions ratio of 2.517, which was estimated from the sequence alignment. *B. garinii* variants found associated with *I. uriae* ticks are shown in bold italics, including two variants (boxed) found in both *I. uriae* and *I. ricinus* ticks. The variants found both in the *I. ricinus* ticks and in the patients with Lyme borreliosis are underlined. Six putative phylogenetic clusters of *B. garinii* genetic variants are indicated. Bar provides the scale for nucleotide distance.

Given the absence of detectable intragenic recombination in the aligned partial *rrs-rrl* IGS locus of *B. garinii*, we carried out phylogenetic analysis of this species' genetic variants from subarctic Eurasia. The corresponding sequence from a representative PBi strain of *B. garinii*, for which whole-genome sequences are publicly available (www.ncbi.nlm.nih.gov/Genomes/index.html), was used as an outgroup. Positions with gaps were excluded from this analysis. Figure 3 is distance phylogram of *rrs-rrl* IGS sequences with bootstrap values of at least 70% for nodes under distance, maximum likelihood and maximum parsimony criteria. All routines produced identical topology of and similar support values for the clades. The phylogenetic analysis defined two monophyletic lineages among the 20 *B. garinii* genetic variants (Figure 3). The first lineage comprised a cluster of two variants of the spirochete found in *I. uriae* on Commander Island. The remaining 18 variants comprised the second lineage composed of 5 clusters, with strong bootstrap support of at least 3 of them. *B. garinii* variants associated with *I. ricinus* ticks predominated in this lineage, including seven variants from patients with Lyme borreliosis which were found in 3 of the 5 clusters.

Notably, the 8 *B. garinii* variants associated with the seabird tick *I. uriae* were found in each of the 6 phylogenetic clusters. In 5 of them, they co-clustered with the 10 *B. garinii* variants found in *I. ricinus* ticks on migratory passerine birds in Scandinavia [1].

## Discussion

This study has built on our previous finding of the association of *B. garinii*, a common cause of neuroborreliosis in Europe, with seabird tick *I. uriae* [4], [23], and apparent overlap of *B. garinii*'s regional enzootic cycles in the northern hemisphere [5], [7]. By quantifying this spirochete's infection of seabirds and *I. uriae* ticks and sequence typing of a representative collection from subarctic Eurasia, the present study was unique in producing the following results: (i) demonstration for the first time of extensive circulation of *B. garinii* in the seabirds and *I. uriae* ticks in the northern Pacific region; (ii) identification of the *rrs-rrl* IGS locus as a typing marker of *B. garinii* strains; (iii) determining complex population structure of *B. garinii*, presumably as a consequence of the spirochete's association with diverse tick vectors and vertebrate reservoirs of its marine and terrestrial natural cycles; and (iv) demonstration of two monophyletic lineages of *B. garinii* strains from subarctic Eurasia, with multiple clusters, each including *I. uriae*-associated variants of the spirochete.

The association of *B. garinii* with seabirds is thought to facilitate this species' wide geographic distribution in the costal regions on both southern and the northern hemispheres [7], and as far as the eastern coast of North America [8]. Frequent infection of *I. uriae* with *B. garinii* on Commander Islands in the northern Pacific Ocean is yet another evidence of this spirochete's global dissemination. Tufted Puffin, a predominant bird species on Toporkov Island, is the primary host of all stages of the *I. uriae* ticks. In agreement with other studies [7], we found a high prevalence of *B. garinii* infection of these ticks.

*B. garinii* strains were genetically more diverse in their *rrs-rrl* IGS sequence, as compared to the equivalent locus of *B. burgdorferi* and *B. afzelii* strains, a finding consistent with previous demonstration of greater heterogeneity the former species' using other markers [10]. Genetic and antigenic heterogeneity of LB *Borrelia* spirochetes presumably is determined by balancing selection under the pressure of their multiple reservoirs' immune responses [30]. *B. garinii*'s preferential association with both multiple passerine and marine bird species may be one determinant of its genetic diversity. On the other hand, relatively high numbers of *B. garinii* variants on Commander Island is unexpected given the island's geographical isolation and presumably few local vertebrate reservoir species, with predominance of Tufted Puffins. Similarly high was the diversity among the few available *B. garinii* samples from *I. uriae* ticks from arctic Norway, Faroe Islands in northern Atlantic and northern Sweden. Overall, the study demonstrated that genetic diversity of local *B. garinii* strains associated with specialist *I. uriae* ticks and few reservoirs is equivalent to regional heterogeneity of this species' strains carried by generalist *I. ricinus* ticks and propagated by multiple mammalian and bird reservoir hosts.

The epidemiological importance of *B. garinii*'s marine enzootic cycle is not known. Humans often exposed to infected seabirds have not been reported to contract LB [23]. Furthermore, seabird isolates of *B. garinii* are sensitive even to low concentrations of human complement (Comstedt et al. submitted). It is possible that seabirds and *I. uriae* ticks contribute to the maintenance of *B. garinii* variants non pathogenic to humans by disseminating them along the coastlines of the continents. On the other hand, the present study demonstrates that the marine and the terrestrial enzootic cycles exchange at least some genetic variants of *B. garinii*. Apparently, the marine and terrestrial natural hosts of *B. garinii* occasionally meet despite the distance and harsh climatic conditions in many seabird colonies, which is a barrier for their encounter and transmission of the spirochete across distinct ecological niches. For example, it has been documented that rodents inhabit some islands in the Baltic Sea where seabirds breed side by side with passerine birds, a setting presumably conducive to the exchange of *B. garinii* strains between different tick species [5]. *B. garinii* isolated from seabirds can also establish a long-term infection in rodents (Comstedt, et al. submitted). Given that passerine birds host *B. garinii* strains found in patients with LB [1], their role as adaptors of this species' variants to infection of humans is plausible. Notably in this respect, each of the six identified phylogenetic clusters of *B. garinii* from diverse sources contained the samples from seabird-associated *I. uriae* ticks. One interpretation of this finding is that the marine enzootic cycle of *B. garinii* serves as a donor of this spirochete's strains, which adapt to terrestrial reservoirs and *I. ricinus* tick.

*B. garinii* variants isolated from patients with LB in southern Sweden were more conserved in their IGS sequence than the variants associated with *I. ricinus* or *I. uriae*. Relatively limited geographical range of these samples was unlikely to account for greater genetic similarity among the isolates, since the same variants were found also in *I. ricinus* ticks from migratory birds or found questing in Sweden and Lithuania. It is possible that *B. garinii* strains causing the disease in humans represent relatively recent clonal expansion of this species. Such expansion of human-pathogenic variant of *B. burgdorferi* represented by prototypic B31 strain is thought to have caused LB epidemic in the northeastern United States [31]. Whereas in the latter case reforestation of North America in the last century may have determined the elimination of the ecological bottleneck [32], factors behind the emergence of the pathogenic variants of *B. garinii* are yet to be identified.

Since Tufted Puffins only come ashore to breed and the rough climate in the Barents Sea area allows only for maximum one molting every year, *I. uriae* ticks may need up to 7 years to complete its developmental cycle [33], [34]. Such slow life cycle may have a negative effect on the proliferation of the spirochetes in the ticks, e.g. decreased cell counts after molting, as observed in this study. The bacteria that remain in the ticks after molting are nevertheless sufficient for maintaining the infection cycle on the isolated island. The high infection prevalence in different stages of *I. uriae* ticks on Comander Islands contrasts the absence of infection of the blood of adult Tufted Puffins, although *Borrelia* spirochetes have been isolated from adult Puffins (*Fratercula arctica*), a close relative of the former bird species [23]. In contrast to the latter study, we did not test the skin, a tissue that is commonly infected by LB group spirochetes, for the infection. In addition, the birds were not subjected to stress, which is implicated in reactivation of latent *B. garinii* infection of birds [35]. Some birds, such as American robins and chickens, are only transiently infected after being challenged with *Borrelia* spirochetes [24], [36]. In contrast, other studies indicate that *Borrelia* spirochetes can persist in some bird species for months [35], [37]. It is possible that only some of newly hatched or fledgling Tufted Puffins in the colony are spirochetemic and transmit the infection to the ticks, which often attack them sitting for weeks in the burrows. Notably, chickens develop resistance to the infection during few weeks after hatching [36]. Therefore, we propose that in a marine natural cycle *B. garinii* survives in *I. uriae* ticks rather than in the birds, a hypothesis testable through examination of fledglings for the presence of *Borrelia* infection.

## Acknowledgments

Dr. Michail Grudinin, Dr. Vladimir Blinov, Dr. Maria Pisareva, Dr. Marina Stukova, Ms. Janna Buzitskaya, from the Institute of Influenza, Russian Academy of Medical Sciences, St. Petersburg, Russian Federation are acknowledged for excellent help with blood sampling. Dr. Nikolay N. Pavlov, Director, Komandorskiy State Nature Biosphere Reserve, Dr. Yuri Artukhin and Dr. Larisa Zelenskaya are commended for their professional guiding and logistic support. We also thank Dr Thomas Jaenson at Uppsala University for species determination of the ticks. Lisette Marjavaara and Elin Nilsson are acknowledged for excellent technical assistance and Betty Guo for critically reading the manuscript. Philippe Hugo Ahlenius at UNEP/GRID-Arendal Maps and Graphics Library is greatly acknowledged for providing us with the map of the Arctic and Subarctic regions. The Swedish Polar research Secretariat is greatly acknowledged for logistic support.

## References

[1] Comstedt P, Bergström S, Olsen B, Garpmo U, Marjavaara L (2006). Migratory passerine birds as reservoirs of Lyme borreliosis in Europe.. Emerg Infect Dis.

[2] Kurtenbach K, De Michelis S, Etti S, Schafer SM, Sewell HS (2002). Host association of *Borrelia burgdorferi* sensu lato–the key role of host complement.. Trends Microbiol.

[3] Kurtenbach K, Peacey M, Rijpkema SG, Hoodless AN, Nuttall PA (1998). Differential transmission of the genospecies of *Borrelia burgdorferi* sensu lato by game birds and small rodents in England.. Appl Environ Microbiol.

[4] Olsen B, Jaenson TG, Noppa L, Bunikis J, Bergström S (1993). A Lyme borreliosis cycle in seabirds and *Ixodes uriae* ticks.. Nature.

[5] Bunikis J, Olsen B, Fingerle V, Bonnedahl J, Wilske B (1996). Molecular polymorphism of the lyme disease agent *Borrelia garinii* in northern Europe is influenced by a novel enzootic *Borrelia* focus in the North Atlantic.. J Clin Microbiol.

[6] Larsson C, Comstedt P, Olsen B, Bergström S (2007). First Record of Lyme Disease *Borrelia* in the Arctic.. Vector Borne Zoonotic Dis.

[7] Olsen B, Duffy DC, Jaenson TG, Gylfe Å, Bonnedahl J (1995). Transhemispheric exchange of Lyme disease spirochetes by seabirds.. J Clin Microbiol.

[8] Smith RP, Muzaffar SB, Lavers J, Lacombe EH, Cahill BK (2006). *Borrelia garinii* in seabird ticks (*Ixodes uriae*), Atlantic Coast, North America.. Emerg Infect Dis.

[9] Gosler A (2006). Birds of the World.

[10] Wilske B, Preac-Mursic V, Gobel UB, Graf B, Jauris S (1993). An OspA serotyping system for *Borrelia burgdorferi* based on reactivity with monoclonal antibodies and OspA sequence analysis.. J Clin Microbiol.

[11] Will G, Jauris-Heipke S, Schwab E, Busch U, Rossler D (1995). Sequence analysis of ospA genes shows homogeneity within *Borrelia burgdorferi* sensu stricto and *Borrelia afzelii* strains but reveals major subgroups within the *Borrelia garinii* species.. Med Microbiol Immunol.

[12] Busch U, Hizo-Teufel C, Boehmer R, Fingerle V, Nitschko H (1996). Three species of *Borrelia burgdorferi* sensu lato (*B. burgdorferi* sensu stricto, *B afzelii,* and *B. garinii*) identified from cerebrospinal fluid isolates by pulsed-field gel electrophoresis and PCR.. J Clin Microbiol.

[13] Roessler D, Hauser U, Wilske B (1997). Heterogeneity of BmpA (P39) among European isolates of *Borrelia burgdorferi* sensu lato and influence of interspecies variability on serodiagnosis.. J Clin Microbiol.

[14] Wang G, van Dam AP, Spanjaard L, Dankert J (1998). Molecular typing of *Borrelia burgdorferi* sensu lato by randomly amplified polymorphic DNA fingerprinting analysis.. J Clin Microbiol.

[15] Tsao JI, Wootton JT, Bunikis J, Luna MG, Fish D (2004). An ecological approach to preventing human infection: vaccinating wild mouse reservoirs intervenes in the Lyme disease cycle.. Proc Natl Acad Sci U S A.

[16] Bunikis J, Garpmo U, Tsao J, Berglund J, Fish D (2004). Sequence typing reveals extensive strain diversity of the Lyme borreliosis agents *Borrelia burgdorferi* in North America and *Borrelia afzelii* in Europe.. Microbiology.

[17] Thompson JD, Higgins DG, Gibson TJ (1994). CLUSTAL W: improving the sensitivity of progressive multiple sequence alignment through sequence weighting, position-specific gap penalties and weight matrix choice.. Nucleic Acids Res.

[18] Maddison DR, Maddison WP (2002). MacClade 4: Analysis of phylogeny and character evolution 4.04..

[19] Rozas J, Rozas R (1999). DnaSP version 3: an integrated program for molecular population genetics and molecular evolution analysis.. Bioinformatics.

[20] Sawyer S (1989). Statistical tests for detecting gene conversion.. Mol Biol Evol.

[21] Jolley KA, Feil EJ, Chan MS, Maiden MC (2001). Sequence type analysis and recombinational tests (START).. Bioinformatics.

[22] Galtier N, Gouy M, Gautier C (1996). SEAVIEW and PHYLO_WIN: two graphic tools for sequence alignment and molecular phylogeny.. Comput Appl Biosci.

[23] Gylfe Å, Olsen B, Strasevicius D, Marti Ras N, Weihe P (1999). Isolation of Lyme disease *Borrelia* from puffins (*Fratercula arctica*) and seabird ticks (*Ixodes uriae*) on the Faeroe Islands.. J Clin Microbiol.

[24] Richter D, Spielman A, Komar N, Matuschka FR (2000). Competence of American robins as reservoir hosts for Lyme disease spirochetes.. Emerg Infect Dis.

[25] Brisson D, Dykhuizen DE (2004). ospC diversity in *Borrelia burgdorferi*: different hosts are different niches.. Genetics.

[26] Bunikis J, Tsao J, Garpmo U, Berglund J, Fish D (2004). Typing of *Borrelia* relapsing fever group strains.. Emerg Infect Dis.

[27] Hanincova K, Taragelova V, Koci J, Schafer SM, Hails R (2003). Association of *Borrelia garinii* and *B. valaisiana* with songbirds in Slovakia.. Appl Environ Microbiol.

[28] Glöckner G, Lehmann R, Romualdi A, Pradella S, Schulte-Spechtel U (2004). Comparative analysis of the *Borrelia garinii* genome.. Nucleic Acids Res.

[29] Bergström S, Olsen B, Burman N, Gothefors L, Jaenson TG (1992). Molecular characterization of *Borrelia burgdorferi* isolated from *Ixodes ricinus* in northern Sweden.. Scand J Infect Dis.

[30] Qiu WG, Bosler EM, Campbell JR, Ugine GD, Wang IN (1997). A population genetic study of Borrelia burgdorferi sensu stricto from eastern Long Island, New York, suggested frequency-dependent selection, gene flow and host adaptation.. Hereditas.

[31] Seinost G, Dykhuizen DE, Dattwyler RJ, Golde WT, Dunn JJ (1999). Four clones of *Borrelia burgdorferi* sensu stricto cause invasive infection in humans.. Infect Immun.

[32] Spielman A (1994). The emergence of Lyme disease and human babesiosis in a changing environment.. Ann N Y Acad Sci.

[33] Sonenshine DE (1993).

[34] Steele GM, Davies CR, Jones LD, Nuttall PA, Rideout K (1990). Life History of the seabird tick, *Ixodes* (*Ceratizodes*) *uriae*, at St. Abb's head, Scotland.. Acarologia.

[35] Gylfe Å, Bergström S, Lundström J, Olsen B (2000). Reactivation of *Borrelia* infection in birds.. Nature.

[36] Piesman J, Dolan MC, Schriefer ME, Burkot TR (1996). Ability of experimentally infected chickens to infect ticks with the Lyme disease spirochete, *Borrelia burgdorferi*.. Am J Trop Med Hyg.

[37] Olsen B, Gylfe Å, Bergström S (1996). Canary finches (*Serinus canaria*) as an avian infection model for Lyme borreliosis.. Microb Pathog.

[38] Bennet L (2005). Erythema migrans in primary health care [Doctoral thesis].

[39] Ornstein K, Berglund J, Nilsson I, Norrby R, Bergström S (2001). Characterization of Lyme borreliosis isolates from patients with erythema migrans and neuroborreliosis in southern Sweden.. J Clin Microbiol.

